# Designer Amyloid Cell-Penetrating Peptides for Potential Use as Gene Transfer Vehicles

**DOI:** 10.3390/biom10010007

**Published:** 2019-12-18

**Authors:** Chrysoula Kokotidou, Sai Vamshi R. Jonnalagadda, Asuka A. Orr, George Vrentzos, Androniki Kretsovali, Phanourios Tamamis, Anna Mitraki

**Affiliations:** 1 Department of Materials Science and Technology, University of Crete, 70013 Heraklion, Grete, Greece; chkokoti@hotmail.com; 2 Institute of Electronic Structure and Laser (IESL) FORTH, 70013 Heraklion, Crete, Greece; 3 Artie McFerrin Department of Chemical Engineering, Texas A&M University College Station, TX 77843-3251, USA; vamshij@tamu.edu (S.V.R.J.); asukaorr@tamu.edu (A.A.O.); 4 Institute of Molecular Biology and Biotechnology (IMBB) FORTH, 70013 Heraklion, Crete, Greece; vrentzos@imbb.forth.gr (G.V.); kretsova@imbb.forth.gr (A.K.)

**Keywords:** computational biology, molecular dynamics, peptide, amyloid, scaffold, gene transfer

## Abstract

Cell-penetrating peptides are used extensively to deliver molecules into cells due to their unique characteristics such as rapid internalization, charge, and non-cytotoxicity. Amyloid fibril biomaterials were reported as gene transfer or retroviral infection enhancers; no cell internalization of the peptides themselves is reported so far. In this study, we focus on two rationally and computationally designed peptides comprised of β-sheet cores derived from naturally occurring protein sequences and designed positively charged and aromatic residues exposed at key residue positions. The β-sheet cores bestow the designed peptides with the ability to self-assemble into amyloid fibrils. The introduction of positively charged and aromatic residues additionally promotes DNA condensation and cell internalization by the self-assembled material formed by the designed peptides. Our results demonstrate that these designer peptide fibrils can efficiently enter mammalian cells while carrying packaged luciferase-encoding plasmid DNA, and they can act as a protein expression enhancer. Interestingly, the peptides additionally exhibited strong antimicrobial activity against the enterobacterium *Escherichia coli.*

## 1. Introduction

The development of biocompatible, efficient, and stable carriers for the delivery of nucleic acids into eukaryotic cells to restore or correct deficient gene products to normal expression levels garnered increased interest in the field of gene therapy. Optimal carriers package the DNA molecule efficiently into a stable complex that can bind and access cells, avoid degradation, and release nucleic acids to the nucleus for gene expression or in the cytosol for gene regulation [1,2,3]. Common hurdles are the inefficient delivery of nucleic acids in their naked form into the cells, due to their strong negative charge that inhibits their internalization, and their susceptibility to nucleolytic enzymes. This led to the development of a variety of non-viral vectors that can incorporate genetic material and efficiently deliver it into the cells [4]. One such vector comprising short cationic peptides called cell-penetrating peptides (CPPs) recently emerged. CPPs have the capacity to effectively cross cellular membranes, have limited toxicity, and could function as transfection carriers for nucleic-acid cargos including small interfering RNAs (siRNAs) and plasmids [5].

CPPs typically consist of 5–30 amino acids rich in arginine and lysine amino-acid groups, which are positively charged due to their protonation at neutral pH. Various energy-dependent or -independent [6] internalization mechanisms were proposed [7], including the interaction of the positively charged residues with the negatively charged phospholipids of the cell membrane to facilitate direct internalization and endosomal uptake via endocytic pathways [8,9]. Due to their ability to penetrate cellular membranes, CPPs could enhance the transportation of conjugated bioactive cargos, which in turn could initiate the expression or function of specific intracellular targets. These bioactive cargos can be conjugated with the CPPs through a covalent bond or through a non-covalent complex formation [4].

The mechanism proposed for the formation of a non-covalent complex and subsequent DNA transduction involves the complexation through electrostatic interaction of the positively charged amino acids of the peptide with the negatively charged nucleic acids and the further internalization of the newly constructed complex into the cell. The complexation of the CPPs with DNA and the subsequent transportation of the peptide–DNA complex through the cell membrane could engender the enhancement of gene transfer, as well as the protection of DNA against enzymatic degradation [10].

Extensive lists of peptide carriers or enhancers of the expression of a gene of interest into cells were proposed that facilitate the non-covalent peptide–DNA complex formation. Among others are the highly cationic TAT peptide that can directly penetrate the plasma membrane as a polyelectrolytic complex upon interacting with plasmid DNA [10], amphipathic α-helical peptide NF55 [11,12], and the PepFect14 peptide vector [13]. Highly cationic peptides, especially polyarginine (nona-arginine, R9) and the T22 peptide (RRWCYRKCYKGYCYRKCR), when fused to the *N*-terminus of GFP along with a His-tag at the C-terminus were recently reported to mediate self-association into nanosized particles that penetrate cells [3,5,6]. These nanosized particles can be exploited for the targeted delivery of both nucleic acids and protein drugs. Very recently, a novel category of promising AMPs was reported, based on a number of bacterial aggregation-prone regions (APRs) which are not toxic to mammalian cells but can induce protein aggregation in the bacterial cell, leading to loss of the bacterial proteostasis and eventual bacterial cell death [14,15]. It seems, therefore, that properly designed protein and peptide aggregates in either synthetic or recombinant form could be amenable to rational design targeting cell penetration and drug or DNA delivery. Cationic, amyloid-forming peptides are also considered promising nanomaterials for boosting gene transduction by utilizing the positive charges on the fibrillar nanosheet formed by the peptides to capture nucleic acids and virion particles and subsequently promote their cell attachment and fusion [16,17,18,19,20].

Amyloid aggregation of initially correctly folded proteins is driven by short amyloidogenic sequence domains within the protein full sequence that self-assemble into fibrils [21,22]. Amyloid formation was thought to be associated solely with amyloid diseases such as Alzheimer’s and Parkinson’s diseases [23,24,25,26]. However, numerous studies demonstrated that amyloids could also be exploited as promising bionanomaterials [27,28] or even assume physiologically relevant roles [29,30].

We previously demonstrated that the ultrashort and homologous peptide sequences GAITIG and GAIIG can spontaneously self-assemble into amyloid fibrils [31,32]. The GAITIG sequence is part of the adenovirus fiber shaft segment that, in the absence of its natural trimerization motif, aggregates into amyloid-type fibrils. By employing a reductionist approach, the sequence GAITIG was previously shown to be a minimal self-assembling building block [32,33]. Similarly, the sequence GAIIG, which is part of the amyloid-β peptide (residues 29–33) and also part of the HIV-1 gp120 V3 loop (residues 24–28), can also spontaneously form a β-sheet amyloid core [31]. Exposed residues, outside the β-sheet GAITIG or GAIIG core, could be accessible and available for suitable selected modifications, rendering the resulting rationally designed sequences available for applications in biomedicine and technology [34,35]. Thus, both peptides were previously used as starting sequences for the computational and experimental design of functional scaffolds [34,35,36].

In the present study, we sought to rationally design self-assembling amyloid peptide sequences containing cationic residues conferring cell-penetrating properties and eventual DNA-binding ability. We employed computational methods, starting with scaffolds YATGAIIGNII and RGDSGAITIGC, and mutated key non-β-sheet positions at the termini of the scaffolds, namely, residues one, two, three, and eleven. We replaced these exposed residue positions with a combination of positively charged residues (Arg, and Lys) and tyrosine residues [37], to mimic the cell-penetrating properties and DNA-binding ability of CPPs. The computational and experimental studies of the two rationally designed amyloid peptides, KYKGAIIGNIK and KYRSGAITIGY (hereinafter referred to as KK and KY, respectively), directed us to three conclusions concerning their properties. Firstly, they can spontaneously self-assemble into amyloid fibrils; secondly, they can interact electrostatically with plasmid DNA to form complexes; thirdly, they can transfer plasmid DNA (pDNA) into mammalian cells and promote protein expression of the gene of interest. Moreover, the formulated peptide/DNA complexes exhibit long-term stability, with very limited cytotoxicity, and the cationic peptides display a strong bactericidal effect against *Escherichia coli*.

## 2. Materials and Methods

### 2.1. Materials

KYKGAIIGNIK and KYRSGAITIGY peptides were custom-synthesized by GenScript with C-terminal amidation. The purity of the peptides was over 95%. The pGL3 plasmid with the SV40 promoter containing the gene for expressing luciferase was obtained from Addgene. The HEK293T cell line was cultured at 37 °C, 5% CO_2_ in DMEM (Gibco) supplemented with 10% fetal bovine serum (Gibco) and 50 μg/mL gentamycin. OPTI-MEM (Gibco) was used to obtain a reduced environment for the optimal transfection conditions. Thiazolyl blue tetrazolium bromide (MTT) and Congo red were purchased from Sigma-Aldrich. Quant-IT PicoGreen double-stranded DNA (dsDNA) assay kit was purchased from ThermoFisher Scientific. The Proteostat^®^ aggresome detection kit was from Enzo.

### 2.2. Computational Methods

We used two separate approaches to rationally design and select two peptides with sequences NH_3_^+^–KYRSGAITIGY–CONH_2_ and NH_3_^+^–KYKGAIIGNIK–CONH_2_. Upon selection of the two peptide sequences, we computationally investigated the two designed peptides using replica exchange molecular dynamics (REMD) simulations. Firstly, we investigated the conformational properties of the isolated, individual designed peptides through infinite dilution REMD simulations. Then, we investigated the self-assembly properties of the designed peptides through finite dilution REMD simulations comprising copies of the designed peptides in a cubic box, per designed peptide. Using the structures extracted from the finite dilution simulations, we categorized the β-sheet structures formed by the designed peptides into two-, three-, four-, five-, and six-stranded antiparallel, parallel, or mixed β-sheet structures, identified the predominant configuration (parallel or antiparallel β-sheets) adopted by each of the two designed peptides, and identified the key β-sheet interactions formed within the self-assembled structures of the two designed peptides. Subsequently, we extracted the highly ordered and well-aligned β-sheet structures, investigated the key interactions formed between the amino acids within the structures, and calculated the solvent accessibility of the designed residues to assess the peptides’ functionality. Below, we provide a detailed description of the computational methods employed to design and study the two peptides.

### 2.3. Rational and Computational Design of the Positively Charged Peptides

We aimed to design functional amyloid peptides with cell-penetrating and DNA-binding properties through two separate approaches.

In the first approach, we used the computationally elucidated structures of elementary β-sheet structural units formed by the amyloid designable scaffold, RGDSGAITIGC [36], as input flexible structural templates to an in-house optimization-based model, which was previously used for the design of functional amyloid materials [34,38]. According to our previous study, the dominant configuration of the β-sheet structures formed by peptide RGDSGAITIGC is antiparallel, and residue positions one, two, three, and eleven are amenable for modification as they are not part of the β-sheet core [36]. In the current study, we used the antiparallel elementary β-sheet structural units formed by the designable scaffold, RGDSGAITIGC, as input to the computational protocol with residue positions one, two, three, and eleven as designable positions (underlined). The computational design model was performed without subjecting the optimization-based model any constraints related to the binding to a specific ion (e.g., materialphore model related constraints as in Reference [34]). Upon solution of the model, a total of 20^4^ sets of mutations were evaluated and ranked according to the energy defined in the objective function. Subsequently, for the post-design constraints, we hypothesized that a combination of positively charged residues (Arg and Lys) and tyrosine residues at the designable positions would be desirable as (i) positively charged residues are known to penetrate cell membranes [39], and (ii) aromatic residues in the order Y > W > F are abundant in proteins interacting with DNA [37]. To introduce cell-penetrating motifs at the N-terminal of the designed sequence, we performed a bioinformatics analysis on the motifs with sequence XXXS, where XXX corresponds to any residues within designable positions one, two, and three, and S corresponds to serine, in cell-penetrating peptides deposited in the Database of Cell-Penetrating Peptides (CPPsite2.0 [40]). From the bioinformatics search, we observed that only RYYS and KYRS sequence motifs occurred as a part of any cell-penetrating peptides. We selected the sequence motif KYRS as we considered that tyrosine at position 3 in the motif RYYS could extend the β-sheet core, probably negatively affecting its functional properties. After imposing the above post-design constraints, the top-ranked designed peptide was the peptide KYRSGAITIGY; thus, we selected the designed peptide with sequence KYRSGAITIGY for further investigation. It is worth noting that the selected designed peptide sequence was among the top 10% of all 20^4^ possible sequences according to the energy ranking defined in the objective function prior to the introduction of any constraints.

In the second approach, we aimed to rationally design a functional amyloid peptide with cell-penetrating and DNA-binding properties using the amyloid designable scaffold, YATGAIIGNII [31], as a basis. As, according to our previous study [31], residue positions one, two, three, and eleven are amenable for modification as they are not part of the β-sheet core, we rationally designed peptide sequences by introducing mutations to the designable (underlined) positions of YATGAIIGNII. Similarly to the rational design described above, we performed a bioinformatics analysis for sequence motifs containing positively charged residues or tyrosines within cell-penetrating peptides deposited in CPPsite2.0 [40]. In the analysis, we disallowed tyrosine at position three to avoid the extension of the β-sheet core of the self-assembled designed peptides. Based on the bioinformatics analysis, the motifs KYK, KYR, RYK, and RYR were possible amino-acid replacements at the designable positions one, two, and three with the motif KYK occurring most frequently. Thus, we mutated the residue positions one, two, and three to lysine, tyrosine, and lysine, respectively. In contrast to the computational design model described above in the first approach, in this approach, motivated by the fact that positively charged residues are beneficial for both cell penetration and DNA binding functionality, we rationally substituted position eleven with lysine. Thus, the aforementioned direction resulted in the designed peptide KYKGAIIGNIK, and we selected the designed peptide for further investigation.

One of the designed peptides was engineered on the basis of NH_3_^+^–RGDSGAITIGC–CONH_2_. For the current study, we investigated both designed peptides with the same NH_3_^+^– and –CONH_2_ terminal ends; we considered that the positive charge of the NH_3_^+^-terminal end could additionally be favorable for the desired DNA-binding and cell-penetrating functional properties of the amyloid materials formed by the designed peptides. Thus, the selected designed peptides with sequence NH_3_^+^–KYRSGAITIGY–CONH_2_ and NH_3_^+^–KYKGAIIGNIK–CONH_2_ were subsequently investigated using infinite and finite dilution simulations, independently, following a protocol developed by Tamamis and Archontis [41], which was initially executed for the study of peptides with sequence NSGAITIG [33], LSFDNSGAITIG [42], and LSGSDSDTLTV [43], and was later widely used by our group to shed light onto the amyloid self-assembly properties of short peptides [31,34,36,38], as described in the sections below.

### 2.4. Infinite Dilution Simulations Investigating the Peptides’ Conformational Properties

We performed infinite dilution simulations of two peptides with sequence KYRSGAITIGY and KYKGAIIGNIK in aqueous solution, independently, using REMD simulations [33,44,45,46,47,48,49] in CHARMM [50]. The REMD simulations consisted of eight replicas with temperatures 283, 300, 318, 336, 356, 377, 403, and 432 K. The temperatures were selected such that the average exchange rates between the different replicas were 27% ± 1% and 28% ± 2% for KYRSGAITIGY and KYKGAIIGNIK, respectively, in line with References [33,41]. The initial structures of the peptides corresponded to linear structures build in CHARMM [50]. The peptides were modeled using the CHARMM19 force field [51], and the aqueous environment was modeled using the FACTS19 [52] implicit solvent model with the surface tension coefficient set to 0.015 kcal∙mol^−1^∙Å^−2^ [31,34,36]. For all simulations, we used Langevin dynamics with a friction coefficient of 5 ps^−1^ introduced on all heavy atoms and simulation snapshots extracted in 100-ps intervals. The duration of each simulation per temperature was 150 ns for both peptides, independently, for an aggregate total simulation duration of 1.2 μs per peptide. Upon completion of the infinite dilution simulations, we extracted the 15,000 simulation snapshots of the 300-K trajectory. We subsequently performed a root-mean-squared deviation (RMSD)-based clustering analysis on the extracted snapshots using Wordom [53,54] and selected representative conformations from the six most populated clusters for each peptide resulting in six conformations per peptide. The clustering analysis was performed based on the backbone atoms of the peptides using a clustering radius of 2 Å and the quality-clustering algorithm. The six extracted conformations per peptide corresponded to the centers, or most representative structure, of each cluster, and they were used as initial structures in the finite dilution REMD simulations investigating the peptides’ self-assembly properties described in the latter section. All six extracted conformations were distinct from the initial extended structures of the two peptides used for the REMD simulations; the RMSDs of the extracted structures of KYRSGAITIGY and KYKGAIIGNIK ranged from 7.4 to 10.6 Å and 7.4 to 8.4 Å, respectively.

### 2.5. Finite Dilution Simulations Investigating the Peptides′ Self-Assembly Properties

We performed independent REMD simulations in CHARMM [50] for both designed peptides to investigate their self-assembly properties, analogously to References [31,33,36,38,41,43]. The simulation system corresponded to six copies of the peptide KYRSGAITIGY in a 157-Å cubic periodic box and KYKGAIIGNIK in a 154-Å cubic periodic box, resulting in an approximately 3 mg/mL concentration for the two simulation systems. The initial conformations of the six peptide copies were obtained from the six conformations extracted from the corresponding aforementioned finite dilution REMD simulations. For each of the two simulations, all six copies of the peptide were initially placed in the center of the cubic periodic box, and then translated by +25 Å in the *x*-direction, −25 Å in the *x*-direction, +25 Å in the *y*-direction, −25 Å in the *y*-direction, +25 Å in the *z*-direction, or −25 Å in the *z*-direction such that a peptide was placed at the center of each face of a 50-Å cubic box. The REMD simulations consisted of 16 replicas with temperatures of 290, 295, 300, 310, 305, 315, 321, 327, 333, 339, 345, 352, 359, 366, 373, and 380 K. The temperatures were selected such that the average exchange rates between the different replicas were 29% ± 2% and 29% ± 2% for KYRSGAITIGY and KYKGAIIGNIK, respectively, in line with References [33,41]. The simulations were performed using the CHARMM19 force field [51] and the FACTS19 implicit solvent model [52] with the surface tension coefficient set to 0.015 kcal∙mol^−1^∙Å^−2^ [31,36,38], with simulation snapshots extracted in 10-ps intervals. For all simulations, we used Langevin dynamics with a friction coefficient of 5 ps^−1^ on all heavy atoms. The duration of each simulation per temperature was 1.000 ns for both peptides, independently, for an aggregate total simulation duration of 16 μs per peptide. Upon completion of the finite dilution simulations, we extracted the 100,000 simulation snapshots of the 300-K trajectory for each peptide independently.

### 2.6. Categorization of β-Sheet Structures and Key β-Sheet Interactions.

**Figure 1:**
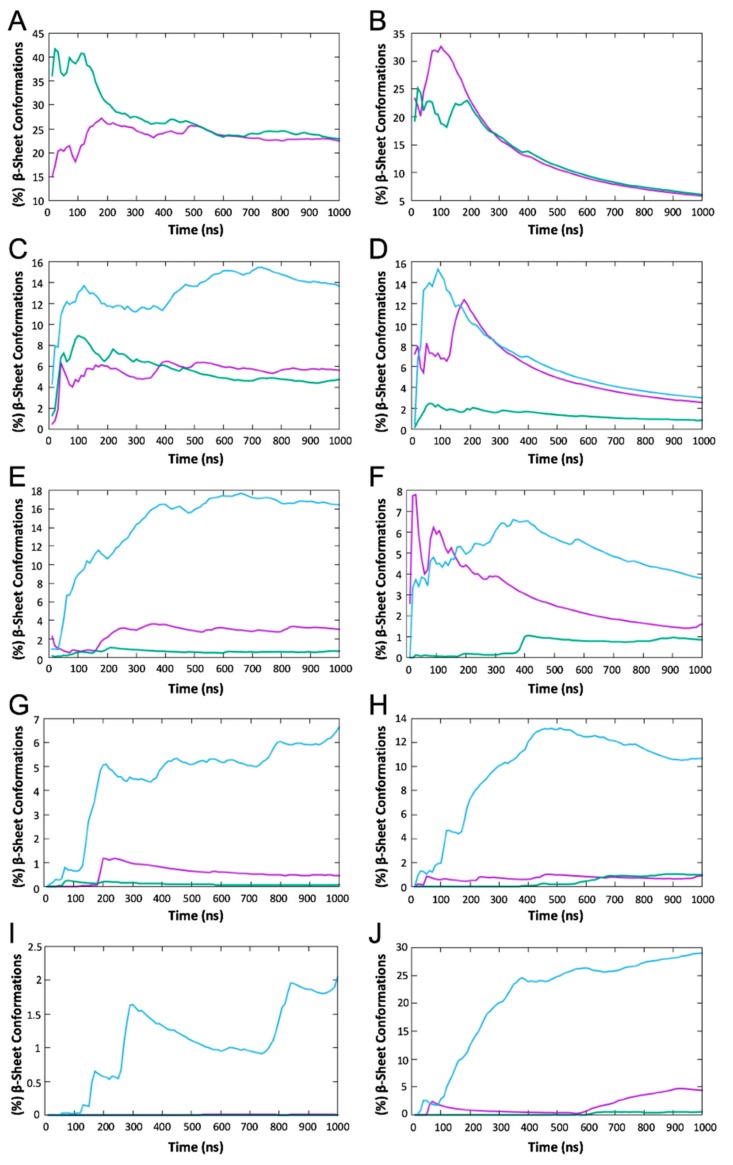
Moving averages of the fraction (%) of conformations with intermolecular β-sheets (*Y*-axis) in the replica exchange molecular dynamics (REMD) simulations at 300 K with respect to time (*X*-axis) for KYRSGAITIGY and KYKGAIIGNIK. Panels (**A**,**C**,**E**,**G**,**I**) correspond to two-, three-, four-, five-, and six-stranded β-sheets of peptide KYRSGAITIGY, respectively. Panels (**B**,**D**,**F**,**H**,**J**) correspond to two-, three-, four-, five-, and six-stranded β-sheets of peptide KYKGAIIGNIK, respectively. Purple corresponds to antiparallel β-sheets, green corresponds to parallel β-sheets, and blue corresponds to mixed β-sheets.

**Figure 2:**
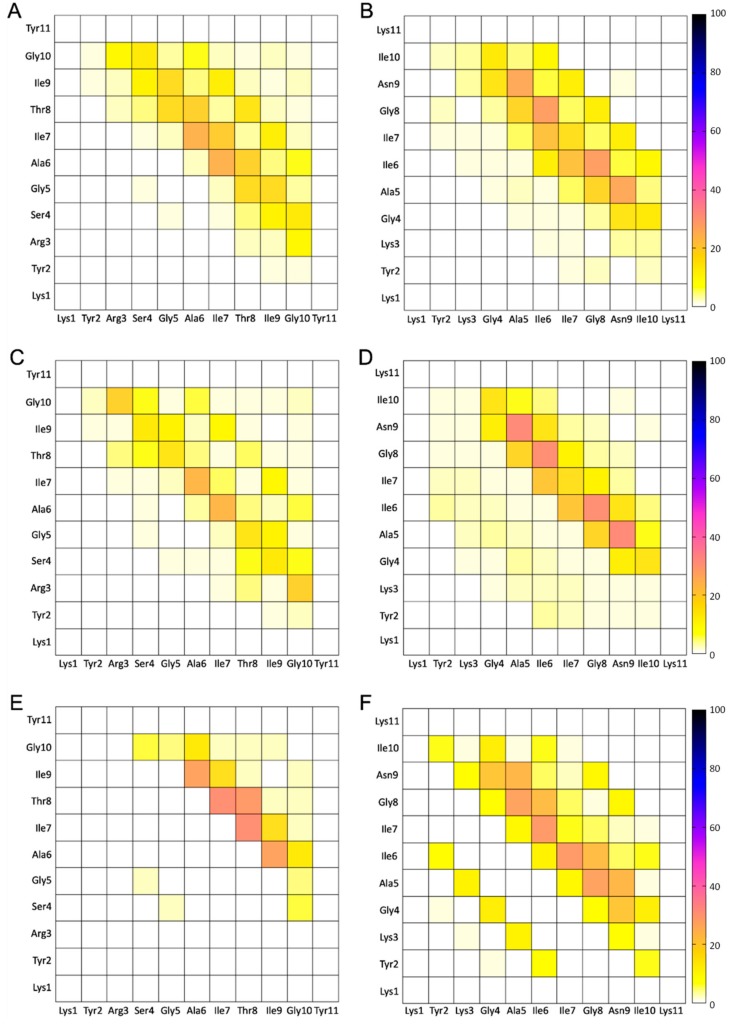
Density (%) maps of residue pairs forming intermolecular β-bridges for KYRSGAITIGY and KYKGAIIGNIK. The pairs of residues belong to nearest neighboring peptides participating in an isolated β-bridge or extended β-sheet conformation in the REMD simulations at 300 K. Panels (**A**,**C**,**E**) correspond to four-, five-, and six-stranded antiparallel configurations of peptide KYRSGAITIGY, respectively. Panels (**B**,**D**,**F**) correspond to four-, five-, and six-stranded antiparallel configurations of peptide KYKGAIIGNIK, respectively.

We identified the formation of intermolecular β-sheet structures and categorized the intermolecular β-sheet patterns into antiparallel, parallel, or mixed two-, three-, four-, five-, and six- stranded β-sheet structures, similarly to References [31,36,38] for designed peptides KYRSGAITIGY and KYKGAIIGNIK. The intermolecular β-sheet structures were identified using DSSP [55] and categorized using in-house FORTRAN programs. We calculated the (%) moving average number of structures containing antiparallel, parallel, or mixed β-sheet structures in each of the two-, three-, four-, five-, and six- stranded peptides as shown in Figure 1. For both designed peptides, the analysis showed that the peptides preferentially formed antiparallel β-sheet structures over parallel β-sheet structures. Following this analysis, we focused our further analysis on four-, five-, and six-stranded antiparallel β-sheet structures, which we considered more complex compared to two- and three-stranded β-sheets [31,33,36,38,41,43]. Thus, we extracted the four-, five-, and six-stranded antiparallel β-sheet structures from each of the simulation trajectories corresponding to the two designed peptides and calculated the (%) probability of a pair of residues belonging to separate adjacent peptides to be involved in a β-bridge conformation. From this analysis, we identified the predominant configuration (parallel or antiparallel) and key patterns of β-sheet interactions, which indicated the key amyloidogenic regions of each designed peptide, as shown in Figure 2. The specific analysis, in addition, also identified if the designed residues predominantly participated in the β-sheet interactions, which would hinder their availability for functionality.

### 2.7. Identification of Well-Aligned and Highly Ordered β-Sheet Conformation Using Polar (P_1_) and Nematic (P_2_) Order Parameters

**Figure 3:**
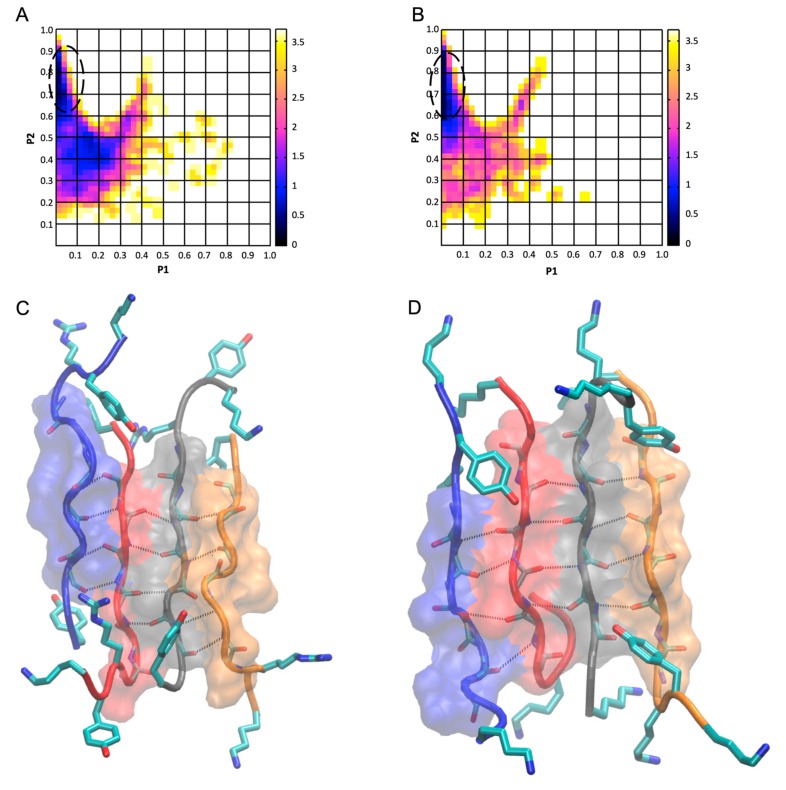
Free-energy surfaces constructed from the two-dimensional (2D) probabilities of order parameters *P*_1_ and *P*_2_ and molecular graphics images of representative structures of KYRSGAITIGY and KYKGAIIGNIK extracted from the free-energy minima. Panels (**A**,**B**) correspond to free-energy surfaces constructed from the 2D probabilities of order parameters *P*_1_ and *P*_2,_ calculated using the four-stranded antiparallel β-sheets formed by KYRSGAITIGY (KY) and KYKGAIIGNIK (KK), respectively, observed in the replica exchange MD simulations at 300 K. The global free-energy minima in the plots are located in basins which are marked using black dashed lines, and, within these, we observe the presence of highly ordered and well-aligned β-sheet conformations. Panels (**C**,**D**) correspond to molecular graphic images of representative highly ordered and well-aligned conformations of peptides KYRSGAITIGY and KYKGAIIGNIK in antiparallel arrangement, respectively. The peptide backbone is shown in tube representation, and the backbone atoms forming β-bridges are shown in thin licorice representation and are colored by name, while the β-bridge-associated hydrogen bonds are shown using black dashed lines. The peptides are colored in blue, red, gray, and orange from left to right. Residue moieties 4–9 in each of the two designed peptides form amyloid zipper-like patterns and are shown in transparent surface representation. Side-chain atoms of residues at positions one, two, three, and eleven are shown in thick licorice representation.

We assessed the extent of peptide alignment and relative orientation of the individual peptides within the four-, five-, and six-stranded antiparallel β-sheet structures using the polar-order *P*_1_ and nematic-order *P*_2_ parameters, calculated through Wordom [53,54]. These parameters are used in the structural characterization of liquid crystals, and they were employed successfully in simulation studies of peptide aggregation [33,41,42,43,56,57]. The analysis was performed in line with References [31,33,36,38,41,43] with the unit vector ${\vec{z}}_{i}$, defined as the segment spanning from the Cα atom of residue four to the Cα atom of residue nine. The analysis was focused on four-, five-, and six-stranded β-sheet structures as they have higher complexity than two- and three-stranded β-sheets; thus, the structural patterns observed in the former can potentially correspond to patterns that may exist in naturally occurring fibrils formed by the peptides. According to the computed P_1_ and P_2_ parameters, among the four-, five-, and six-stranded antiparallel β-sheet structures, highly populated and highly ordered β-sheet structures were observed only in the four-stranded antiparallel β-sheet structures for both peptide systems. This can be attributed to the limited number of peptides within the finite dilution self-assembly simulations. Thus, as four-stranded β-sheets are complex enough to correspond to elementary β-sheet structural units and are present within a sufficient number of simulation snapshots for statistical analysis, we focused our analysis on four-stranded antiparallel β-sheet structures formed by the two designed peptides and constructed free-energy landscapes (Figure 3A,B) for each peptide system using two-dimensional probability *P*(*P*_1_,*P*_2_) and Equation (1).

(1) $$G\left(P_{1}{,P}_{2}\right)={-k}_{B}T\ln[P\left(P_{1}{,P}_{2}\right)].$$

From the free-energy basins within the free-energy landscapes, we extracted the highly ordered and well-aligned β-sheet structures for each peptide system. Representative structures of highly ordered and well-aligned antiparallel β-sheet structures of KYRSGAITIGY and KYKGAIIGNIK are shown in Figure 3C,D, respectively.

Given that simulations are used with the expectation of reproducing experimental structures and dynamics, the reaction coordinates used for the construction of free-energy landscapes [58], in tandem with the choice of simulation scheme and solvent, are crucial. Regarding the former, the individual and combined uses of *P*_1_ and *P*_2_ parameters as reaction coordinates in free-energy landscapes to study conformations of amyloid peptides, to the best of our knowledge, were first carried out by Cecchini et al. [56] and Tamamis et al. [33] respectively. These parameters were shown to constitute effective reaction coordinates to construct free-energy landscapes through which the most highly ordered and well-aligned β-sheet conformations of amyloid peptides can be extracted from the simulations [31,33,34,36,38,41,43]. At the same time, such free-energy landscapes are valuable given that simulations can efficiently capture the conformational space of the simulated peptides. For this purpose, regarding the latter, we used REMD simulations [44,45,46,47,48,49] coupled with implicit solvation provided through FACTS19 [52]. The coordinate exchange achieved through a Metropolis criterion in REMD simulations allows low-temperature replicas to escape local minima and to borrow the sampling efficiency of high-temperature replicas, while it ensures that energetically inaccessible conformations (at a given temperature) are not allowed, and that all replicas gain access to conformations with the proper thermodynamic weight [41]. In addition, and most importantly for studies involving peptide self-assembly, the high-temperature runs permit the frequent dissolution and re-organization of aggregates and the sampling of a wide variety of intermolecular structures [41]. This is an essential feature of the REMD simulations, which a traditional run at room temperature would lack [41]. Specifically, in this study, the following temperatures were used: 290, 295, 300, 305, 310, 315, 321, 327, 333, 339, 345, 352, 359, 366, 373, and 380 K, which in both simulated systems allowed a highly uniform exchange probability of 29% ± 2% [41], as mentioned above. The fairly constant and relatively high exchange rate throughout the temperature space was considered important for observed aggregates at the experimental temperature to be relatively stable, to enable the exploration of intermolecular structures involving several peptides, and also allow the aggregates to undergo sufficient re-organization at higher temperatures [41]. The relatively high exchange rate was especially considered beneficial for replicas to execute random walks covering the entire temperature space [41]. In conjunction with the REMD simulation scheme, the use of implicit solvation, specifically FACTS19, which was used extensively in several studies [31,32,33,34,36,38,41,42,43,59,60,61,62], can significantly lower the computational cost and accelerate peptide conformational transitions, while simultaneously providing a compromise between accuracy and efficiency [41]. Nevertheless, the choice of an implicit solvent model needs to be conducted with caution, and it should be verified either with respect to explicit solvent results and/or with respect to experimental studies [41].

### 2.8. Determination of Exposure of Designed Residues

We determined the solvent accessibility of the designed residues to assess their exposure and their potential functionality. The degree of solvent accessibility of residues at positions one, two, three, and eleven within the extracted highly ordered and well-aligned four-stranded antiparallel β-sheet structures for both designed peptides, KYRSGAITIGY and KYKGAIIGNIK, was determined using Wordom [53,54]. Similarly to References [34,36], we calculated the solvent accessible surface area, i.e., the total accessible surface area ratio of residues at positions one, two, three, and eleven of the two central peptides in each highly ordered and well-aligned four-stranded antiparallel β-sheet structures. As the degree of solvent accessibility of the two outer peptides was artificially high due to the absence of interacting peptides on both sides, their values were not considered in the analysis. The total accessible surface areas were defined as the maximum solvent accessible surface area (SASA), which was the SASA of the set of residues with all other atoms removed, as in References [34,36].

### 2.9. Amyloid Fibril Formation

Peptide powders were dissolved in sterile filtered double-distilled water in a final concentration of 6 mg/mL. The resulting peptide solutions were incubated at room temperature for three days. Formation of fibrils was confirmed by field-emission SEM (FE-SEM), TEM, and Congo red staining as previously described [36].

### 2.10. Field-Emission Scanning Electron Microscopy (FE-SEM)

After the three-day incubation period, 10 μL of each peptide sample, diluted 1:6, was deposited on a cover glass and was air-dried overnight. Dried samples were covered with 10 nm of Au/Pd sputtering. Observation experiments were performed using a JEOL JSM-7000F microscope operating at 15 kV.

### 2.11. Transmission Electron Microscopy (TEM)

Each sample (5 μL) was deposited directly onto a formvar/carbon-coated electron microscopy grid for 2 min. Excess was carefully removed with a filter paper, and the sample was stained with 2% *w*/*v* uranyl acetate for 2 min. The observations were conducted with a JEOL JEM-2100 transmission electron microscope at 80 kV.

### 2.12. Congo Red Staining

Each peptide solution (20 μL) was thoroughly mixed with 5 μL of a fresh Congo red assay solution (10 mM Congo Red, 2.5 mM NaOH in 50% ethanol). A drop of the mixture was deposited onto a glass coverslip and was examined before or after drying at room temperature, with a Zeiss Stemi 2000-C microscope with and without the use of a crossed polarizer.

### 2.13. Formulation of Cationic Peptides and pDNA Complexes

The pGL3-SV40 luciferase-expressing plasmid was mixed with the self-assembled peptides at various ratios, in a total volume of 50 μL of double-distilled sterile water. Formulations were allowed to assemble for at least 1 h in room temperature before cell transfection.

### 2.14. Gel Retardation Assay

The formation and DNA condensation of the CPP–DNA complexes was verified by electrophoresis on a 0.5% agarose gel in TAE (Tris Acetate-EDTA) 1× buffer and imaged by staining of the gel with Gel-Red (Biotium). Complexes were formed as previously described. Firstly, 0.5 μg of pGL3-SV40 was mixed with 10, 25, 50, 100, or 200 μg of each peptide for 1 h. Then, 4 μL of loading buffer was mixed with the samples before the electrophoresis.

### 2.15. PicoGreen Fluorescence Quenching Experiments

The pDNA (0.5 μg) was labeled with 30 μL of the PicoGreen reagent (1:150 *w*/*v*) in TE (Tris-EDTA) buffer for 30 min at room temperature. The labelled pDNA was mixed with 10, 25, 50, 100, and 200 μg of the self-assembled peptides for 1 h at room temperature. PicoGreen binds to the grooves of the DNA backbone and strongly fluoresces when excited at 488 nm. Quenching of the fluorescence indicates packaging of the nucleic acid. The quenched fluorescence was analyzed in a BioRad CFX Connect Real-Time System, and the naked pDNA labeled with the PicoGreen fluorescence signal was used to normalize the signal detected from the peptide–DNA signal. Values were expressed as quenching percentage.

### 2.16. Zeta Potential Measurement

The pDNA–peptide complexes were formulated in a final volume of 200 μL of filtered double-distilled water in different DNA–peptide concentration ratios (1:312, 1:625, 1:1250, 1:2500, and 1:5000). An additional dilution with 1 mM NaCl to up to a 1-mL volume followed before measurement. Measurements were performed in a ZetaSizer Malvern instrument, using the Smoluchowski model, set to a number of five runs.

### 2.17. Cell Internalization of the Amyloid-Forming Peptides

Firstly, 5 × 10^4^ HEK293T human embryonic kidney cells were seeded for 24 h in a 24-well plate after addition of a 13-mm Tissue Culture coverslip at the bottom of the well. The culture medium (DMEM) was replaced, and 25 μg of the fibrillar peptide sequence, diluted in 0.5 mL of DMEM, was added for overnight incubation. the culture media were aspirated, and the cells were carefully washed two times with PBS 1×. The cells were fixed with 4% formaldehyde for 30 min, washed with PBS 1×, and permeabilized for 5 min with 0.5% Triton X-100, 3 mm EDTA, pH 8. After washing twice with PBS 1×, the cells were treated with the staining solution containing the Proteostat^®^ aggresome staining and Hoechst nuclear dye. Additional washes with PBS 1× followed, the coverslip was mounted on a microscope coverslip, and the internalization efficacy of the peptides was assessed in a Leica SP8 inverted confocal microscope at excitation/emission 500/600 nm for the Proteostat^®^ dye and excitation/emission 350/461 nm for Hoechst 33342.

### 2.18. MTT Cell Proliferation Assay

Cell viability in the presence of the peptides was studied by monitoring the conversion of thiazolyl blue tetrazolium bromide reagent (MTT) into formazan by the mitochondrial dehydrogenases of the living cells. HEK293T cells with concentrations of 7 × 10^3^ cells/well were cultured in a 96-well plate for 24 h. Removal of the medium was followed by treatment of the cells with increasing concentrations (10, 25, 50, 100, 200 μg) of the self-assembled peptide, suspended in a total volume of 200 μL of culture medium. Cells that were not treated with the peptide served as control. After an incubation period of 48 h, the medium was carefully removed and replaced with 100 μL of fresh medium and 10 μL of MTT (5 mg/mL) dissolved in PBS 1×. The cells were incubated for 4 h to allow the development of the purple formazan products and the MTT/culture medium was substituted with 100 μL of an isopropanol–DMSO 1:1 solution. The formazan crystals were allowed to dissolve for 15 min at 37 °C. The absorbance was measured at 570 nm in a Synergy HTX BioTEK Plate Reader.

### 2.19. Plasmid Transfection and Luciferase Assay

HEK293T cells were seeded in a 24-well plate at a density of 6 × 10^4^ cells per well and grown until 60% confluency. The cultured medium was smoothly removed, and 500 μL of OPTI-MEM medium containing 50 μL of the preformed pDNA–peptide complexes in ratios (1:312, 1:625, 1:1250, 1:2500, and 1:5000 after sequential dilutions) were carefully added into the wells. After a 4-h incubation period at 37 °C, the medium was removed, 1 mL of fresh DMEM supplemented with FBS and gentamycin was added, and the cells were incubated for 48 h to allow for reporter gene expression. Luciferase activity was measured by using a luciferase assay system according to the manufacturer’s guidelines (Bright-Glo, Promega, Madison, WI, USA) in a Synergy HTX BioTEK Plate Reader. Protein concentration in cell lysates was assessed by the Bradford assay.

### 2.20. Antimicrobial Testing

The antimicrobial activity of the peptides was assessed by following the broth microdilution method [63] adjusted to the guidelines of CLSI (Clinical and Laboratory Standards Institute. Performance standards for antimicrobial susceptibility testing; 16th informational supplement. CLSI document M100-S16CLSI, Wayne, PA (2006)) and EUCAST (European Committee for Antimicrobial Susceptibility Testing) of the European Society of Clinical Microbiology and Infectious Diseases (ESCMID). Bacteria were grown in Luria Bertani broth (LB) in a shaking incubator at 37 °C overnight, using individual colonies retrieved from a fresh overnight BL21 DE3 plate. The bacterial suspension was adjusted to 10^6^ CFU/mL in LB according to the MacFarlane standard. Then, 50 μL of the bacterial inoculum was mixed in 96-well plates with the twofold diluted peptide solutions (50 μL) to reach final concentrations of peptides ranging from 0.75 mg/mL to 0.005 mg/mL, including two inhibition controls (kanamycin 50 μg/mL and ampicillin 100 μg/mL), a sterility control, and a growth control. Each peptide concentration was tested in triplicate. The plate was incubated for 24 h at 37 °C, and the optical density was measured at 600 nm (OD_600_) in a Synergy HTX BioTEK Plate Reader.

### 2.21. pDNA Internalization and Sub-Cellular Localization

Firstly, 7 × 10^4^ HEK293T human embryonic kidney cells were seeded for 24 h in a 24-well plate after addition of a 13-mm TC Coverslip at the bottom of the well. The following day, 50 ng of pGL3-SV40 plasmid was incubated for 30 min with the PicoGreen dye (1:150 dilution), and it was further mixed with 250 μg of the KK or KY peptide for 1 h. The medium was carefully removed, and 50 μL of the pre-stained pGL3-SV40–peptide complex diluted in 500 μL of OPTI-MEM was added to the well. After a 4-h incubation period at 37 °C, the culture media were aspirated, and the cells were carefully washed two times with PBS 1×. The cells were fixed with 4% formaldehyde for 15 min, washed with PBS 1×, and permeabilized for 5 min with 0.1% Triton X-100. After washing twice with PBS 1× and incubating for 30 min with BSA 2%/PBS 1×, the cells were treated with the staining solution containing the AlexaFluor 680 phalloidin dye. Two additional washing steps with PBS 1× followed before the coverslip was mounted on a microscope coverslip containing a drop of the DAPI nuclear staining dye. The internalization and subcellular localization of the stained pDNA was assessed in a Leica SP8 inverted confocal microscope at ex/em 679/702 nm for the AlexaFluor 680 phalloidin dye, ex/em 488/520nm for the PicoGreen dye, and ex/em 360/460 nm for the DAPI nucleus stain.

## 3. Results

### 3.1. Categorization of β-Sheet Structures and Key β-Sheet Interactions

We investigated the self-assembly properties of designed peptides KYRSGAITIGY and KYKGAIIGNIK using independent REMD simulations. Within the simulations, the peptides frequently formed β-sheet structures comprising two-, three-, four-, five-, and six-stranded β-sheet structures (Figure 1). Two-stranded β-sheet structures were observed in both parallel and antiparallel conformations (Figure 1). Three-, four-, five-, and six-stranded β-sheet structures were observed in parallel, antiparallel, and mixed conformations (Figure 1). As in References [24,26,28,39,45,46], structures comprising mixed conformations, in which the peptides formed at least one pair of parallel and one pair of antiparallel β-sheet conformations, or complex conformations, in which one peptide formed β-sheet interactions with more than two separate peptides, were not investigated as they are not expected to be representative of naturally occurring amyloid fibrils. In line with the dominant configuration of the designable scaffolds that were used to produce the designed peptides sequences, both peptides showed overall higher tendency to form antiparallel β-sheet structures over parallel β-sheet structures (Figure 1C–J). We focused our subsequent investigation on antiparallel β-sheets which appeared to be predominantly formed. Thus, we extracted the antiparallel four-, five-, and six-stranded β-sheet structures for further analysis, as they are more likely to represent naturally occurring fibrils than the less complex two- and three-stranded β-sheet structures.

Using these structures, we calculated the propensities of any two residues belonging to separate adjacent β-sheet bonded peptides to form intermolecular β-bridge interactions (Figure 2). In the designed peptide KYRSGAITIGY, the residues involved in β-bridge interactions were predominantly within the GAITIG motif (Figure 2A,C,E). In the designed peptide KYKGAIIGNIK, the residues involved in β-bridge interactions were predominantly within the GAIIGN motif (Figure 2B,D,F). Importantly, for both peptides, the designed residues were not involved in β-bridge interactions, indicating that the designed residues may be exposed and possess functional properties, including cell penetration and DNA binding.

### 3.2. Identification of Well-Aligned and Highly Ordered β-Sheet Conformation Using Polar (P_1_) and Nematic (P_2_) Order Parameters

We determined the degree of alignment and order of the peptides within the four-, five-, and six-stranded antiparallel β-sheet structures based on *P*_1_ and *P*_2_ parameters. For both of the designed peptides, KYRSGAITIGY and KYKGAIIGNIK, similarly to our previous studies [31,34,36], highly ordered and well-aligned antiparallel β-sheet structures predominantly occurred within four-stranded, rather than within five- or six-stranded, antiparallel β-sheet structures, potentially due to the larger population of four-stranded antiparallel β-sheet structures. Thus, we constructed free-energy landscapes based on the *P*_1_ and *P*_2_ parameters corresponding to the four-stranded antiparallel β-sheet structures (Figure 3A,B). From the free-energy basin, indicated by black dotted lines in Figure 3A,B, we extracted the highly ordered and well-aligned four-stranded antiparallel β-sheet structures, as these structures can potentially correspond to the elementary β-sheet structural units of the naturally occurring fibrils. Representative conformations of the four-stranded antiparallel β-sheet structures are shown in Figure 3C,D. The ensembles of highly ordered and well-aligned four-stranded antiparallel β-sheet structures for both the designed peptides were collected, and they are analyzed below to determine the functionality of the designed residues.

### 3.3. Structural Analysis of the Four-Stranded Highly Ordered and Well-Aligned Structures

In the four-stranded highly ordered and well-aligned antiparallel β-sheet structures of the designed peptides, the amyloid steric zipper comprised residues GAITIG for KYRSGAITIGY and residues GAIIGN for KYKGAIIGNIK, in line with our previous analysis (Figure 2). Apart from this, we observed that the designed residues of both peptides rarely formed specific interactions with neighboring residues, but rather formed infrequent interactions with nearby residues; thus, such interactions are not analytically described here.

We determined the degree of solvent accessibility of the designed residues within the highly ordered and well-aligned four-stranded antiparallel β-sheet structures to assess their exposure and, thus, their potential functionality. The degree of solvent accessibility of the designed residues is presented in Table 1. For both of the designed peptides, KYRSGAITIGY and KYKGAIIGNIK, the degree of solvent accessibility of all four designed residues, with respect to the initial scaffolds, was above 0.40. According to our previous study on a peptide with sequence NH_3_^+^–RGDSGAITIGC–CONH_2_, the solvent accessibility of rationally designed Cys was 0.34 ± 0.09, which was experimentally shown to be adequately exposed for potential metal binding properties [36]. Thus, the designed residues of the two peptides of the current study should be considered adequately solvent-exposed for DNA-binding and cell-penetrating functionality.

Combined, the sufficient degree of exposure of the designed residues and the low instances of the designed residues interacting with other residues within the highly ordered and well-aligned four-stranded antiparallel β-sheet structures support that the designed residues could potentially yield cell-penetrating and DNA-binding properties. It is important to note here that that the CPP properties are expected to be an outcome of the exposed residues displayed at the surface of the amyloid fibril, since the peptides self-assemble into fibrils and do not exist as individual molecules.

### 3.4. Designer Peptides KK and KY Self-Assemble into Positively Charged Amyloid Fibrils

**Figure 4:**
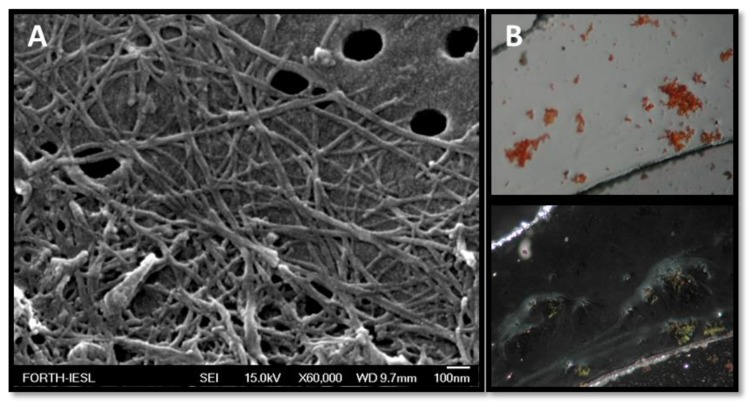
(**A**) Field-emission SEM (FE-SEM) picture of the self-assembled peptide NH_3_^+^–KYKGAIIGNIK–CONH_2_ (KK) after incubation in sterile double-distilled water for three days. Distinct fibrillar morphology is observed. FE-SEM scale bar = 100 nm. (**B**) Congo red staining confirms the formation of amyloid fibrils due to the yellow-green birefringence under crossed polarizer. Top: without the use of a crossed polarizer; bottom: with the use of a crossed polarizer. Magnification = 30×.

**Figure 5:**
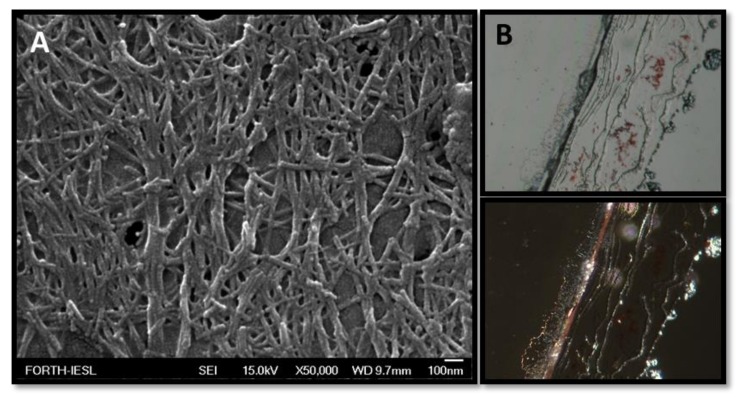
(**A**) FE-SEM picture of the self-assembled peptide NH_3_^+^–KYRSGAITIGY–CONH_2_ (KY) after incubation in sterile double-distilled water for three days. Distinct fibrillar morphology is observed. FE-SEM scale bar = 100 nm. (**B**) Congo red staining confirms the formation of amyloid fibrils due to the yellow-green birefringence under crossed polarizer. Top: without the use of a crossed polarizer; bottom: with the use of a crossed polarizer. Magnification = 30×.

**Figure 6:**
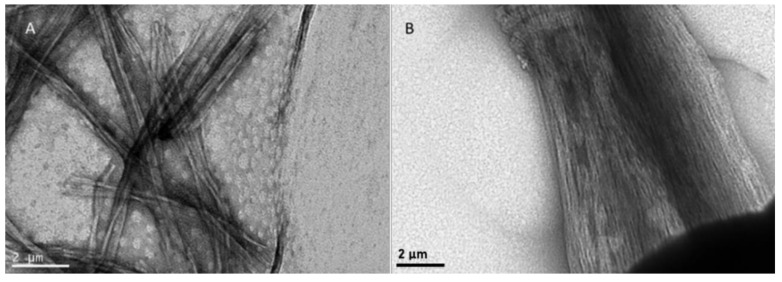
TEM micrographs of the NH_3_^+^–KYKGAIIGNIK–CONH_2_ peptide fibrils negatively stained with 2% uranyl acetate (**A**) before incubation with plasmid DNA (pDNA), and (**B**) after incubation with pDNA for 1 h in a 1:1000 DNA/peptide ratio. Change in the conformation of the fibrils after binding with pDNA, from a random orientation to a more aligned configuration with adherent fibrils, suggests DNA binding. Scale bar = 2 μm.

**Figure 7:**
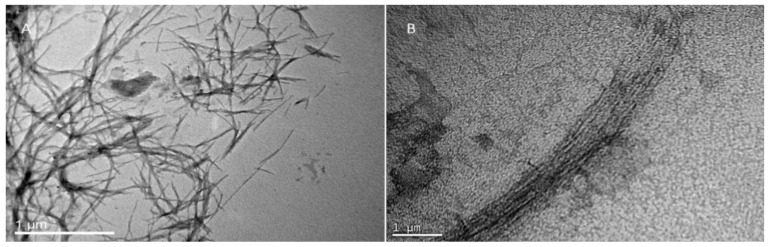
TEM micrographs of the NH_3_^+^–KYRSGAITIGY–CONH_2_ peptide fibrils negatively stained with 2% uranyl acetate (**A**) before incubation with pDNA, and (**B**) after incubation with pDNA for 1 h in a 1:1000 DNA/peptide ratio. Change in the conformation of the fibrils after binding with pDNA, from a random orientation to a more aligned configuration with adherent fibrils, suggests DNA binding. Scale bar = 1 μm.

Both peptides NH_3_^+^–KYKGAIIGNIK–CONH_2_ (KK) and NH_3_^+^–KYRSGAITIGY–CONH_2_ (KY) were designed to contain a beta-sheet-forming core, GAIIG and GAITIG, respectively, that was reported to self-assemble into amyloid fibrils in vivo and in vitro [31,32]. To assess if our beta-sheet-containing sequences corresponded to amyloid-like morphologies after self-assembly, FE-SEM and TEM observations were performed. We further validated that the peptides can self-assemble into amyloid fibrils with Congo red, one of the most commonly used dyes for amyloid detection. Both peptide powders, after being dissolved in sterile deionized water, acquired a fibrillar morphology of long, straight, randomly oriented fibrils with widths in the range of 10–20 nm as observed under FE-SEM (Figure 4A and Figure 5A) and TEM (Figure 6A and Figure 7A). Moreover, the addition of Congo red stain to the incubated peptides uncovered the amyloid nature of the fibrils due to the yellow-green birefringence of the peptides under a crossed polarizer (Figure 4B and Figure 5B). A combination of positively charged residues (Arg and Lys) at the designable positions on both scaffolds resulted in a positive zeta potential, indicating the cationic peptide KK at 32.3 ± 0.91 mV and KY at 31.2 ± 2.19 mV (Table 2).

### 3.5. Amyloid Cationic Peptides Package pDNA by Forming Stable Fibrillar Complexes

To examine the binding and condensation ability of our amyloidal peptides with pDNA, gel retardation, a PicoGreen fluorescence assay, and TEM observations were performed.

**Figure 8:**
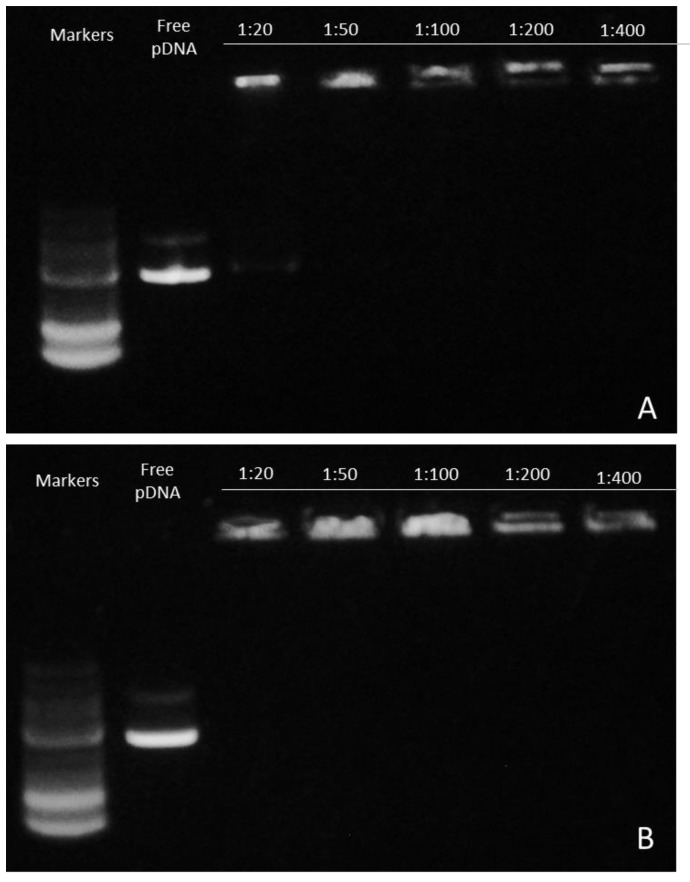
Gel retardation assay for the binding efficiency of the amyloidal peptides with pDNA. Peptides (**A**) KK and (**B**) KY were complexed with 500 ng of pGL3-SV40 in pDNA/peptide ratios varying from 1:20 to 1:400 for 30 min. Formed complexes were run on a 0.5% agarose gel. Free pDNA was used as control.

The pDNA complexes were prepared with the two cationic peptides at DNA/peptide ratios ranging from 1:20 to 1:400. After a 1-h incubation period, they were analyzed for changes in their mobility by a 0.5% agarose gel electrophoresis. Both peptides bound and completely retarded pDNA in the wells (Figure 8). It should also be noted a slight decrease in the intensity of the Gel-Red stain fluorescence at the highest concentrations of the KK peptide complexes, suggesting further fluorescence quenching due to the additional pDNA association. The aim of this experiment was to identify the DNA condensation capacity of the peptides, since plasmid DNA has a highly negative charge and each of the peptide monomers can contribute positive charges attributed to the arginine and lysine residues and also to the unprotected amino group at the N-terminal end. Concluding the above results, we verified that our peptides can fully package pDNA.

**Figure 9:**
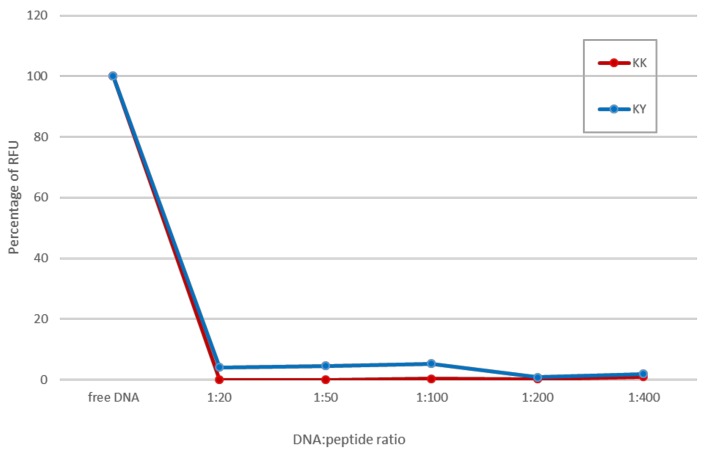
Percentage of pDNA binding to the amyloidal peptides according to PicoGreen fluorescence quenching assay. NH_3_^+^–KYKGAIIGNIK–CONH_2_ (red) and NH_3_^+^–KYRSGAITIGY–CONH_2_ (blue) were mixed with a constant concentration of pDNA (500 ng) in various ratios for 30 min. Complexes were further stained with the PicoGreen dsDNA dye. The fluorescence intensity of the complexes was then measured and normalized compared to the fluorescence intensity of the PicoGreen bound to free pDNA. The percentage of free and unbound pDNA was measured according to the fluorescence quenching level. Low fluorescence percentages indicate DNA binding to the peptides and subsequent quenching of the fluorescence signal.

As an additional confirmation for the binding of the peptides to pDNA, a PicoGreen fluorescence quenching assay was conducted. In the assay, pDNA was mixed with the PicoGreen dye that emits a strong fluorescent signal when excited at 488 nm. Quenching of this signal indicates packaging of the DNA and subsequent binding. Comparing the remaining fluorescence after the complex formation with the fluorescence of the naked pDNA, the percentage of the fluorescence loss corresponds to the level of DNA packaging by the peptides. Complexes of pDNA with the peptides achieved maximal quenching of the PicoGreen signal in all of the DNA–peptide ratios tested (Figure 9), which confirms that, even at low concentrations, our amyloid cationic peptides possess a high binding capacity, a result that is in agreement with the gel retardation assay experiment.

### 3.6. DNA Binding Alters the Morphology of the Fibrils

We next investigated the effect of DNA binding on the morphology of the fibrils after incubation. Interestingly, while amyloid peptides KK and KY exhibited a randomly oriented architecture (Figure 6A and Figure 7A), when these peptides were incubated with the pDNA molecules, they seemed to adhere to each other, affording a more aligned assembly (Figure 6B and Figure 7B). This bundling of the fibrils could be mediated by the electrostatic attraction between negatively charged pDNA molecules and cationic residues belonging to adjacent fibrils/beta sheets.

### 3.7. Long-Term Stability of the pDNA/Peptide Complexes

Moreover, KK–DNA and KY–DNA complexes displayed excellent stability in long-term storage (over four months) at room temperature or in refrigerated conditions without the use of any additives. Furthermore, after lyophilization of the peptide–DNA complex solutions and subsequent resuspension in water, no apparent loss of their binding capability and stability was observed, after testing with the aforementioned methods (results not shown).

### 3.8. Internalization of the Cationic Peptides and pDNA–Peptide Complexes and Their Subcellular Localization

**Figure 10:**
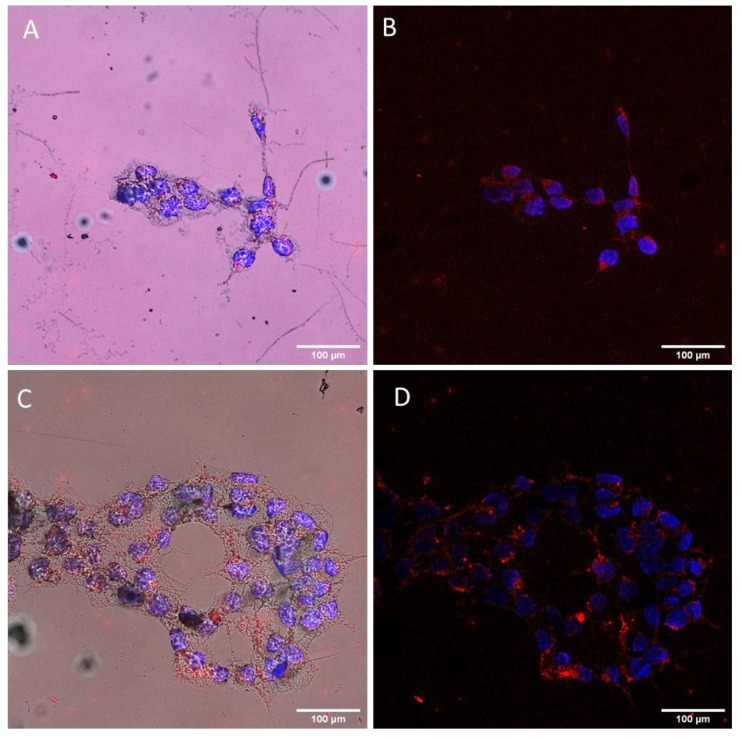
Cellular uptake of the cationic amyloidal peptides. HEK293T cells were exposed to 25 μg of KK and KY peptide, followed by a 24-h incubation. For confocal microscopy observations, cells were washed and stained with a mixture of the Proteostat^®^ dye that binds to the amyloid fibrils and Hoechst 33,342 for nuclear stain. Pictures (**A**,**B**) correspond to the KK peptide, and pictures (**C**,**D**) correspond to the KY peptide. For pictures (**A**,**C**), the bright-field illumination form was applied additionally, to circumscribe the limits of the cell membranes. Moreover, z-stacks for each peptide were obtained to ensure the internalization and not the external cell adhesion of the peptides (results not shown). Scale bar = 100 μm.

**Figure 11:**
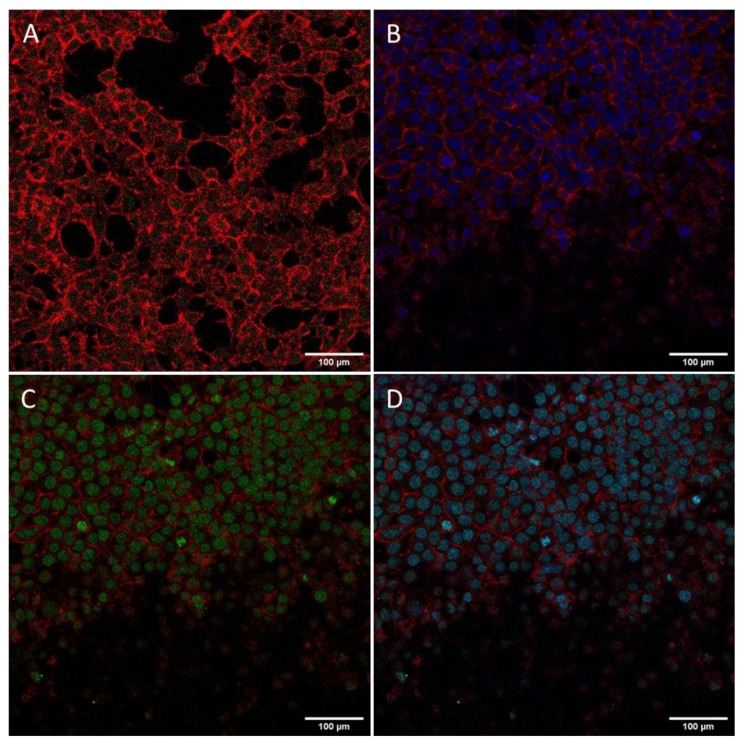
pGL3-SV40 delivery into the nucleus with the aid of the KK peptide. HEK293T cells were treated for 4 h with a pre-stained PicoGreen pDNA/KK complex at a 1:5000 ratio. PicoGreen dye without adding the DNA/peptide complex was used as a control. Cells were washed and stained with AlexaFluor680 phalloidin for visualizing cell membranes (red) and DAPI for visualizing the nucleus (blue). Confocal image of the cells (**A**) incubated only with the PicoGreen stain control, (**B**) after incubation for 4 h with the complex applying only the DAPI (blue) and phalloidin (red) filter, and (**C**) with the PicoGreen filter for pDNA visualization (green) and phalloidin (red). (**D**) A merged picture with the three stains (all filters applied) confirms the successful transportation of the pGL3-SV40 into the nucleus. KY rendered similar results. Scale bar = 100 μm.

As concluded in the computational analysis of the two peptides, the designed cationic residues have a high probability of being exposed and accessible for cell penetration and DNA binding. We examined the cell internalization propensity of the two peptides by employing the Proteostat^®^ staining assay. The PROTEOSTAT^®^ dye is a red fluorescent molecular rotor dye, which specifically intercalates into the cross-beta spine of protein and peptide structures. This binding inhibits the dye rotation and leads to a strong fluorescence emission [64]. Peptides KK and KY were incubated with HEK293T cells and were subsequently stained with the Proteostat^®^ dye. Cell were also stained with the nuclear Hoechst dye to better distinguish the cell nucleus location. Both peptides seemed to internalize in the cell and to localize in the cytoplasm (Figure 10). For additional internalization detection studies concerning the pDNA–peptide complexes, kidney embryonic cells HEK293T were incubated with the pre-stained peptide–pGL3 DNA complexes for 4 h and subsequently imaged in a confocal fluorescent microscope. We detected the pre-stained plasmid with Picogreen mainly in the nuclear area (Figure 11), indicating successful transfection and transition of pDNA in the nucleus within 4 h with the use of the peptide carriers. Figure 11A shows only the PicoGreen stain control. PicoGreen, despite being able to stain free dsDNA, is not considered a cell-permeable dye. To confirm that the green fluorescence was attributed predominantly to the pre-stained pDNA inserted in the nucleus, and not PicoGreen interference, cells were incubated for 4 h only with the PicoGreen stain in the same concentrations used for staining of the pDNA. Only a dim green fluorescence was observed in the cell nucleus, indicating that PicoGreen unspecific staining did not interfere with our observations. Cells incubated with the pre-stained pDNA/peptide complex are shown in Figure 11B, where only the cell nucleus (blue) and actin (red) were visualized, whereas, in Figure 11C, the PicoGreen (green) and actin (red) filters were applied. An intense green fluorescence signal in the nucleus was detected in Figure 11C, indicative of the effective internalization and transfer of the pre-stained SV40–pDNA into the nuclear area. Eventually, when all the filters were applied, the cell nucleus acquired a blue/green color attributed to the mixed color of the DAPI and PicoGreen fluorescence signals (Figure 11D).

### 3.9. Transfection Studies of the Cell-Penetrating Amyloid Peptide–pDNA Complexes

**Figure 12:**
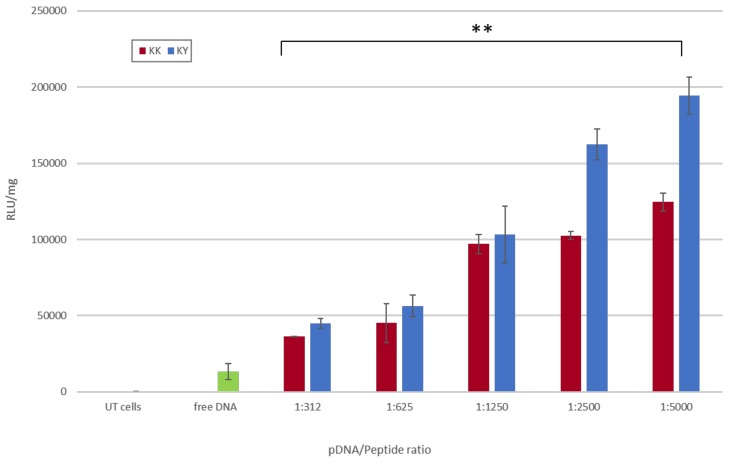
Levels of expression of the luciferase protein in the HEK293T cells after transfection with the pGL3-SV40/KK and pGL3-SV40/KY complexes at different ratios for 4 h and a subsequent incubation period of 48 h. Untreated cells and free pDNA were used as controls. ** denotes the significant difference in the RLU (Relative Light Units)/mg between the untreated cells and the transfected cells (*p* < 0.01).

We investigated the ability of our amyloid peptides to deliver the luciferase-expressing plasmid pGL3-SV40 into the cells and trigger effective gene expression. For gene expression studies, kidney embryonic cells HEK293T were incubated with the peptide/DNA complexes (Table 3) for 48 h, followed by a luciferase activity detection assay. The KK–pDNA and KY–pDNA complexes mediated detectable and satisfactory transfection levels of the pGL3-SV40 plasmid as detected by the luciferase expression levels (Figure 12). KK–DNA and KY–DNA complexes had their highest transfection efficacy at a DNA/peptide ratio of 1:5000, while decreasing the peptide concentration to a 1:312 ratio led to decreased transfection efficacy for both peptides. This can be attributed to the higher overall positive charge of the complexes in ratios where the peptide concentration was increased. As depicted in Table 2, KK and KY complexes started from a 1:5000 DNA/peptide ratio with a positive zeta potential of 27.45 ± 0.9 mV and 28.8 ± 0.21 mV, respectively. Keeping a constant DNA concentration of 50 ng and gradually decreasing the peptide concentration from 250 μg to 15.6 μg, the DNA/peptide ratio decreased to 1:312 and resulted in a decreased zeta potential of 1.95 ± 1.02 mV and 4.2 ± 0.74 mV for the KK–pDNA and KY–pDNA complexes, respectively. The results indicate that there is a direct correlation between the amount of higher positively charged peptide complexes and the transfection levels. Moreover, it should be noted that KY–DNA complexes achieved higher luciferase gene expression level compared to KK–DNA complexes.

### 3.10. Designed Peptides Are Toxic to Bacteria But Not to Mammalian Cells

**Figure 13:**
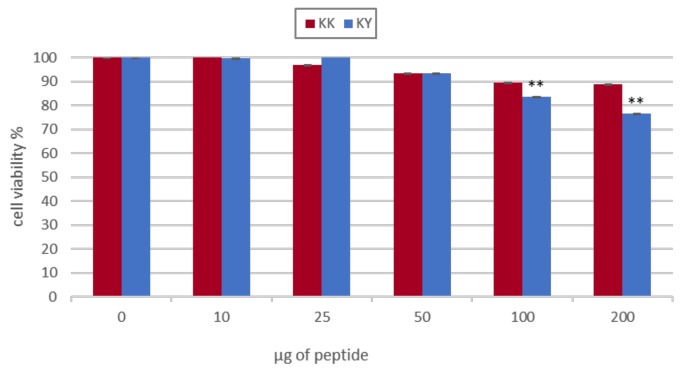
Thiazolyl blue tetrazolium bromide (MTT) cell viability assay results of the amyloid peptides incubated for 48 h in HEK293T cells. KK peptide is represented with red, and KY peptide is represented with blue. Various concentrations of the peptides were used varying from 10 to 200 μg. Results are expressed as a percentage value of control cells cultured without the addition of peptides (control = 100%). ** denotes the significant difference in cell viability between the untreated cells and the transfected cells with the peptides (*p* < 0.01).

**Figure 14:**
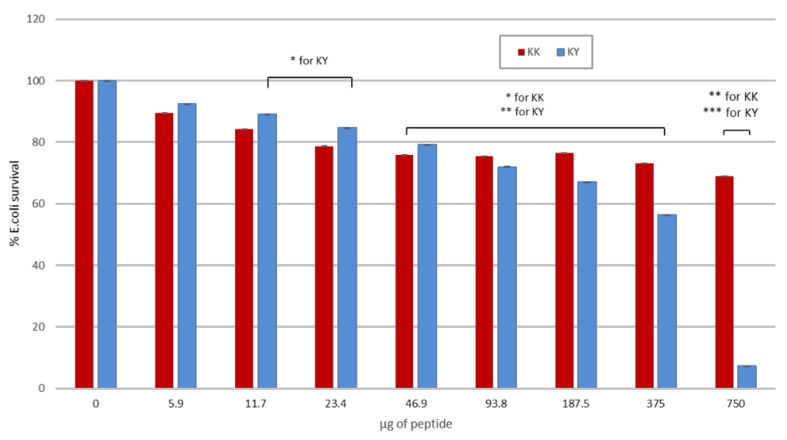
Antimicrobial activity of peptides KK (red) and KY (blue) against *Escherichia coli* bacteria after treatment with increasing concentrations of the peptides. The cultures of bacteria were treated for 24 h in 37 °C, followed by absorbance measurements at 600 nm. Untreated cultures (0 μg of peptide) were used as control, and the measurements were performed in triplicate. * *p* < 0.05, ** *p* < 0.01, *** *p* < 0.0001.

An ideal gene delivery vehicle exhibits very limited or no cytotoxicity to the cells being transfected. Indications of a toxic carrier are initially assessed by cell viability assays such as the MTT test. To confirm that the designed peptides have limited or no cytotoxicity, we carried out transfections with the KK and KY peptides at increasing concentrations and measured the impact on the cell line using the MTT assay. No significant cytotoxicity related to KK and KY peptides was observed; therefore, the peptides can be considered non-toxic to the cell line in the conditions tested (Figure 13). We further examined the antimicrobial potency of our cationic amyloid peptides against a common Gram-negative bacterial species, *E. coli* BL21 DE3. It should be noted that both cationic peptides KK and KY inhibited bacterial population growth. However, KY had better antimicrobial activity by reducing the bacterial population to 50% at concentrations around 0.375 mg/mL compared to KK, which required higher concentrations for the same result (Figure 14).

## 4. Discussion

In the present study, we employed computational methods toward the design of amyloid cell-penetrating biomaterials with DNA-binding functionalities. The computational design was based on the amyloid scaffolds YATGAIIGNII [31] and RGDSGAITIGC [36], which contain the β-sheet cores GAIIG and GAITIG, and which were mutated at key non-β-sheet positions at their termini, namely, at residue positions one, two, three, and eleven. We hypothesized that inserting positively charged residues (Arg and Lys) at these specific positions would favor cell penetration and DNA binding, as recorded in similar studies [65,66]. We purposely left the N-terminal end unprotected for potential involvement of the positively charged amino group in the electrostatic interactions of the peptide. Moreover, aromatic residues in the order Y > W > F are abundant in proteins interacting with DNA [37]. Thus, we hypothesized that tyrosine residues at key positions would additionally favor DNA interaction with our scaffolds.

To elucidate if the novel peptide sequences NH_3_^+^–KYKGAIIGNIK–CONH_2_ and NH_3_^+^–KYRSGAITIGY–CONH_2_ (KK and KY, respectively) had the desired self-assembly properties, we independently investigated the designed peptides using REMD simulations, which revealed that they can spontaneously self-assemble, predominantly in antiparallel β-sheets. Importantly, the designed residues in both scaffolds were not involved in β-bridge interactions, suggesting that these residues are exposed and could possess cell-penetrating and DNA-binding properties. The amyloid nature of KK and KY was further experimentally verified when the peptides, dissolved in sterile filtered double-distilled water, formed the characteristic amyloid fibrils as assessed by TEM, FE-SEM, and Congo red staining.

Important characteristics should be taken into consideration when utilizing positively charged peptides for transferring cargo into cells, especially for gene therapy purposes: (1) effective binding of the oligonucleotide of interest, (2) efficient cellular membrane translocation of the peptide, (3) transfer of the CPP conjugate into the cell and subsequent release of the oligonucleotide, and (4) very limited or no cytotoxicity at all.

The common characteristic of cationic CPPs is their positive net charge, which originates from their basic residues, arginine and lysine. The positive net charge plays a pivotal role in mediating the internalization of the naked peptide and a variety of therapeutic cargoes into mammalian cells. TAT-derived peptides [67], synthetic polyarginines [68], and penetratin [69] are among the most effective representatives of the cationic class of cell-penetrating peptides. Both designer peptides exhibited a positive zeta potential, with 32.3 ± 0.91 mV for the KK peptide and 31.2 ± 2.19 mV for the KY peptide, confirming the exposure of the arginine and lysine residues and also classifying the peptide assemblies as positively charged.

CPP internalization can be attributed to various mechanisms. Routes can be divided into two broad categories: direct penetration of the plasma membrane through interaction of the positively charged peptides with the negatively charged membrane components and phospholipid bilayer [6] or through energy-dependent endocytic pathways, especially when peptides are associated with cargo molecules. Moreover, the internalization process can be dependent on various factors, for example, concentration of the peptide, and properties of the cargo molecules or the cell line [4]. Amyloid fibrils possess cell adhesive properties and can mimic the fibrillar morphology of the extracellular matrix through functionalization with cell-adhesive ligands [70,71]. Moreover, positively charged amyloid nanosheets can also be used as a “docking station” for DNA condensation and retroviral transduction enhancement [16,17,20]. However, due to their extended fibrillar morphology, they were not considered until now as possible candidates for cell internalization and gene carriers. In the present study, the designed peptides successfully achieved non-cytotoxic internalization into the HEK293T cells after an incubation period of 24 h, as revealed by MTT and Proteostat aggresome staining. However, the exact cell uptake mechanisms remain to be elucidated.

For a facile gene transfer application, the peptide carrier should be able to bind and protect the genetic material effectively. Positively charged peptides can interact with the negatively charged phosphate backbone of DNA through electrostatic interactions, leading to condensation of the nucleic-acid biomolecules [72]. DNA-condensing, cell-penetrating peptides must retain their condensation abilities following cell internalization in order to prolong the life of the carried nucleic acid [73] and prevent DNA degradation by cytosolic nucleases [74]. Generally, following endocytosis, a significant amount of internalized DNA is targeted to the lysosomes where it is degraded [75]. Even the remaining “free” DNA has a life expectancy of 50–90 min in the cytoplasm since it is subject to degradation by nucleases [73]. The presence of a carrier that can protect the DNA integrity is essential for safe pDNA transition into the nucleus. Cationic peptides KK and KY were incubated with the luciferase-carrying plasmid, and their DNA-binding abilities were assessed by gel retardation, a PicoGreen assay, and TEM. Since amyloid fibrils cannot diffuse through the agarose pores during electrophoresis due to their morphology, any observed delay in the pDNA migration reflects the extent of the peptide DNA-binding ability. In order to test the DNA-binding capacity of the peptides, we used a high pDNA concentration relative to the peptide concentration. Full retardation of the plasmid band was observed in all of the DNA/peptide ratios tested. Furthermore, the PicoGreen quenching assay showed that, when peptides were mixed with the pre-stained pDNA in the same complex ratios, full quenching of the fluorescent signal was observed. Thus, the gel retardation and fluorescence quenching results point to the DNA condensation ability of the peptides. Interestingly, TEM observation of the peptide–pDNA revealed a change in the arrangement of the fibrils from a randomly oriented fibrillary morphology to bundled assemblies that seem to be connected to each other. We speculate that this alteration is a result of electrostatic interactions between the negatively charged DNA and the positively charged residues emanating from different fibrillar moieties.

**Figure 15:**
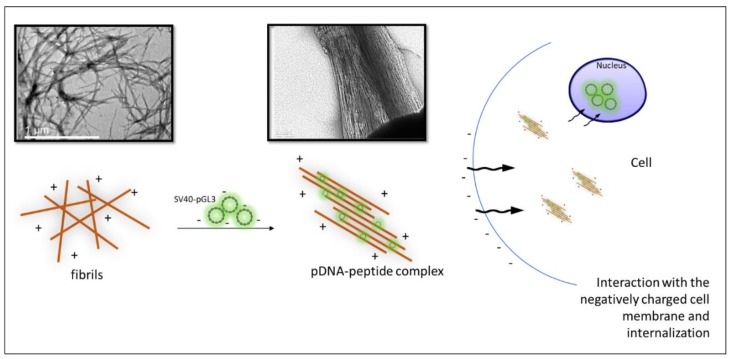
Schematic representation of the pDNA/peptide complex formation and cell uptake through electrostatic interactions. Objects are not drawn to scale.

Peptide transfection studies showed efficient gene delivery results by the pDNA/KK and pDNA/KY complexes in HEK293T cells after a minimal incubation period of 4 h. The delivery efficiency was significantly increased with the increase in peptide concentration. For both peptides, the zeta potential of the complexes increased, from 1.95 ± 1.02 to 27.45 ± 0.9 for pDNA/KK and from 4.2 ± 0.74 to 28.8 ± 0.21 for pDNA/KY (corresponding to DNA/peptide ratios from 1:312 to 1:5000). Therefore, as the added peptide in the complex increased, the DNA negative charge was completely neutralized, and positive charges were in excess. Concurrently, the transfection efficiency increased along with the increase in the overall charge complex from 1:312 to 1:5000. It was reported that the excess of positive charge in the transfecting DNA/peptide complex leads to a more efficient gene transfer. The positive charges may serve in part as a means for electrostatic binding and condensation of DNA [76], and in part for the translocation through the negatively charged anionic phospholipids and/or for the neutralization of various charge factors on the cell surface. It is plausible to conclude that, as the negative charge of the complexes was reduced, the transduction efficiency increased, resulting in a direct correlation of the pDNA/peptide complex charge and the gene delivery efficacy. Furthermore, KY–DNA complexes achieved higher luciferase gene expression levels compared to KK–DNA complexes. This cannot be attributed solely to the difference in overall charge, since the zeta potential measurements fluctuated in the same levels. Arginine-containing sequences may have an advantage in rapid cell penetration due to the ability of the guanidine head to form bidentate hydrogen bonds with the negatively charged membrane constituents [77]. Recent theoretical and experimental studies also point to an important role of arginines in CPP peptides, allowing the peptides to self-associate, thereby enhancing their transduction and potentially their bioavailability [78]. The confocal observations of cells treated with the pre-stained SV40-pGL3 plasmid show that it entered the nucleus in a period of 4 h, as observed from the blue/green overlay on the nuclear area. Figure 15 summarizes the steps leading to the delivery and expression of pDNA by the peptides studied in this work.

Generally, CPPs are designed for noninvasive cargo transport, but they can also possess specific antibacterial activity. Such examples of antimicrobial CPPs are [65] TP-10 [79], MAP [80], TAT [81], penetratin [80,82], pVEC [80], and ε-poly-l-lysines [83], all of which can act as antibacterial agents depending on the peptide concentration and the composition of the bacterial membrane. A proposed mechanism of the cell-penetrating peptides’ antimicrobial activity is the carpet-like model, according to which the positively charged domain of the peptides interacts with and binds to the negatively charged phospholipids on the bacterial membrane, covering the cell surface in a carpet like manner. When a certain concentration of the peptide is reached, the membrane is locally destabilized, allowing the passage of the peptides through different perturbation mechanisms [7]. Peptides NH_3_^+^–KYKGAIIGNIK–CONH_2_ and NH_3_^+^–KYRSGAITIGY–CONH_2_ are both cationic, cell-penetrating peptides which can self-aggregate into amyloid fibrils. They also exhibited bactericidal activity against the *E. coli* strain tested. The peptide NH_3_^+^–KYRSGAITIGY–CONH_2_ required a concentration of 0.375 mg/mL to reduce the bacterial population to 50%, compared to NH_3_^+^–KYKGAIIGNIK–CONH_2_, which required a higher peptide concentration. Cationic aggregating peptides comprising arginine residues, as well as short aggregation-prone residue stretches, were recently identified via bioinformatics approaches in the bacterial proteome and were reported to internalize in mammalian cells. They were also found to be lethal to bacterial cells [14,15]. Their specific toxicity toward bacterial and not mammalian cells involves uptake mechanisms and specific cross-aggregation with bacterial homologous sequences; this cross-aggregation subsequently leads to disruption of bacterial protein homeostasis as a result of the accumulation of protein aggregates [14,15]. Elucidating the mechanism of antimicrobial action of the two peptides reported here will be the subject of future studies. It did not escape our attention that the quasi-homologous sequence SAIIGI was identified with a high cross-aggregation score with *E. coli* proteins in Reference [14], and the designed RSAIIGIIRRPRSAIIGIIRR sequence was predicted to have antibacterial action [14]. It is plausible to hypothesize that the antibacterial activity of the two peptides reported here could follow the same mechanism as that reported in Reference [14], i.e., a cell-penetrating activity mediated by positively charged residues and cross-interaction of the amyloid cores with bacterial sequences, leading to aggregates that disrupt bacterial proteostasis. However, the exact mechanism remains to be elucidated in future studies.

## 5. Conclusions

In this study, we rationally designed two functional peptides that combine amyloid fibril characteristics and beneficial cell penetrating properties. The novel peptide sequences comprised the fibrillar β-sheet core of peptides GAIIG and GAITIG and self-assembled spontaneously into amyloid fibrils when dissolved in water. Through incorporation of the positively charged amino acids arginine and lysine and the aromatic residue tyrosine at carefully selected residue positions, we succeeded in mimicking the cell internalization, as well as DNA-binding and -condensing abilities, of previously in-depth tested cell-penetrating peptides. More importantly, the DNA/peptide fibrillar assemblies were able to transfect and enhance the luciferase protein expression in a charge- and peptide concentration-dependent manner. Moreover, these peptides were able to effectively and drastically decrease *E. coli*’s culture population under the conditions tested.

Amyloid-forming peptides are gaining increasing importance in biomedical applications, including tissue engineering, nanovaccine engineering, and biosensing [70,84]. We anticipate that our designer amyloid materials could constitute a stepping stone for utilizing amyloids as novel biomaterial scaffolds that combine cell penetration and gene transfer with antibacterial properties. Future studies could focus on combining additional properties to the currently designed materials, such as the transfer of siRNAs and protein therapeutic cargos, as well as examining and improving their antimicrobial activity for different bacteria and microbes. Amyloid-forming CPPs present advantages compared to traditional CPPs, such as the possibility to engineer different delivery functionalities onto the same self-assembling scaffold. Moreover, the cross-seeding between the amyloid-forming CPPs and other amyloid peptides, a well-known property of amyloidogenic proteins and peptides [85,86,87], could potentially be exploited toward multifunctional biomaterial design.

## Figures and Tables

**Table 1:** Degree of solvent accessibility of the four designed residues in the two designed peptides KYRSGAITIGY and KYKGAIIGNIK. The four designed residues in each peptide are in bold. The degree of solvent accessibility was calculated for the side chains of the designed residues based on the definitions provided in References [34,36].

Peptide	Residue Position 1	Residue Position 2	Residue Position 3	Residue Position 11
NH_3_^+^–**KYR**SGAITIG**Y**–CONH_2_	0.58 ± 0.06	0.45 ± 0.10	0.49 ± 0.08	0.43 ± 0.11
NH_3_^+^–**KYK**GAIIGNI**K**–CONH_2_	0.63 ± 0.06	0.42 ± 0.09	0.53 ± 0.06	0.53 ± 0.07

**Table 2:** The average zeta potential of the cationic amyloid peptides. pDNA—plasmid DNA.

Zeta Potential (mV)		
pDNA/Peptide Ratio	NH_3_^+^–KYKGAIIGNIK–CONH_2_	NH3^+^–KYRSGAITIGY–CONH_2_
Peptide only	32.3 ± 0.91	31.2 ± 2.19
1:5000	27.45 ±0.9	28.8 ± 0.21
1:2500	26.9 ± 0.67	24.1 ± 1.25
1:1250	24.9 ± 0.66	14.8 ± 1.72
1:625	22.1 ± 2.92	13.7 ± 2.75
1:312	1.95 ± 1.02	4.2 ± 0.74

**Table 3:** Summary of the peptide and pDNA concentrations used for the formation of the complexes.

pDNA/Peptide Ratio	Peptide Concentration (μg/mL)	pDNA Concentration (ng/μL)
1:5000	250	50
1:2500	125	50
1:1250	52.5	50
1:625	31.2	50
1:312	15.6	50
